# 中国肺癌发病死亡的估计和流行趋势研究

**DOI:** 10.3779/j.issn.1009-3419.2010.05.20

**Published:** 2010-05-20

**Authors:** 万青 陈, 思维 张, 小农 邹

**Affiliations:** 100021 北京，全国肿瘤防治研究办公室 National Ofce for Cancer Prevention and Control, Beijing 100021, China

**Keywords:** 肺肿瘤, 发病率, 死亡率, 流行病, 肿瘤登记, 中国, Lung neoplasms, Incidence, Mortality, Epidemiology, Cancer registry, China

## Abstract

**背景与目的:**

本研究旨在探讨我国肺癌的流行特征，对发病和死亡进行估计。

**方法:**

对全国肿瘤登记中心收集的全国各肿瘤登记处1988年-2005年18年间上报的肺癌发病和死亡登记的数据资料进行整理。利用2004年和2005年全国第三次死因回顾抽样调查数据中肺癌的死亡率，结合同时期肿瘤登记数据的死亡发病比，估计目前我国肺癌的发病率。选取数据较为齐全的北京、上海、湖北武汉、黑龙江哈尔滨、河北磁县、江苏启东、浙江嘉善、广西扶绥、福建长乐、河南林州10个登记处的资料，通过肺癌发病率的年变化率计算，分析18年间肺癌流行趋势。

**结果:**

2005年我国肺癌新发病例数为536 407人，死亡病例数475 768人，通过对10个登记处18年发病死亡数据分析，登记点肺癌发病率呈现逐年上升趋势，年平均增长1.63%，但年龄调整后每年降低0.55%。

**结论:**

肺癌是影响我国居民健康的主要恶性肿瘤，发病和死亡呈现逐年上升趋势，主要的原因是人口老龄化导致的，必须采用积极有效的防治措施。

## Abstract (en)

**Background and objective:**

The aim of this study is to analyze lung cancer epidemiological trend and estimate lung cancer burden in China.

**Methods:**

Lung cancer age specifc mortality and incidence rate ratios in di?erent areas and sexes were obtained from national cancer registration database in 2004 and 2005. Cancer crude mortalities were retrieved from the database of the third national death survey, 2004-2005. Age specifc incidence rates of lung cancer were calculated using mortality and M/I ratios. Annual percent change (APC) was estimated by log regression model using Joint Point sofware by analyzing pooled lung cancer incidence data from 10 cancer registries from 1988 to 2005.

**Results:**

The total estimated new cases and deaths of lung cancer in 2005 were 536 407 and 475 768 which were higher in male than in female. Tere was 1.63% increase of lung cancer incidence per year from 1988 to 2005, however, the trend showed a slowdown by 0.55% annually afer adjusted by age.

**Conclusion:**

Lung cancer is one of major health issues in China and the burden is geting serious. Ageing population is main cause for increasing incidence and mortality of lung cancer. Effective cancer prevention and control is imperative. Especially, tobacco control should be carried out in statewide.

肺癌是常见的恶性肿瘤，根据全国肿瘤防治研究办公室调查^[1]^统计，20世纪70年代中期开展的全国第一次死因回顾调查表明，我国肺癌死亡率为5.47/10万，在癌症死因中，排在胃癌、食管癌、肝癌及宫颈癌之后，居第5位，占全部癌症死亡7.43%。与同期其它国家的肺癌死亡率水平相比，处于较低水平。90年代初的全国第二次死因抽样调查^[2]^结果中，肺癌死亡率为17.27/10万，居癌症死因第3位，仅次于胃癌和食管癌。21世纪，卫生部开展了第三次死因回顾调查^[3]^发现，肺癌的死亡率明显升高。

由于人口老龄化的加剧，经济快速增长带来的工业化、城市化进程的高速发展而导致的环境恶化，以及吸烟率居高不下，肺癌的危害正逐渐显现。作为我国重要的恶性肿瘤之一的肺癌，已经被卫生部列为今后癌症防治的重点^[4]^。然而，由于肿瘤登记系统尚不健全，肺癌的负担情况还不清楚，为癌症防治策略的制定带来阻碍。此研究旨在通过目前现有的登记资料和死因调查结果，通过科学的统计方法对我国目前的肺癌发病死亡及趋势进行评估，为基础临床和病因学研究提供可靠的信息。

## 1 材料与方法

### 1.1 资料来源

本研究资料取自以及全国第三次死因回顾抽样调查数据中160个具有全国代表性的死因监测点（其中2个调查点因为质量问题被去除）的不同地区、性别、年龄的肺癌死亡资料，以及《中国部分市、县恶性肿瘤的发病与死亡》第1、2、3卷和2003年、2004年和2005年肿瘤登记年报中肺癌的发病、死亡和人口数据。在目前45个登记处的资料基础上，选取2004年和2005年资料完整性可靠性较好的32个登记处资料进行汇总整理。1988年-2005年间长期资料齐全的10个登记处的资料进行合并^[5-10]^，分别是北京、上海、武汉、哈尔滨、河北磁县、江苏启东、浙江嘉善、广西扶绥、福建长乐、河南林州，以分析登记地区汇总的肺癌发病率和死亡率的变化。

### 1.2 统计学方法

数据均采用ICD10编码。根据不同地区性质，分为农村地区和城市地区。年龄组划分为0岁-29岁、30岁-34岁、35岁-39岁………80岁-84岁和85岁以上共13个年龄组。利用2004年和2005年汇总登记资料拟合泊松回归（Poisson regression）模型，考虑不同地区、不同性别估算肺癌年龄别死亡发病比（age-specific mortality incidence ratio），结合全国第三次死因调查肺癌死亡率和2005年1%人口抽样调查人口数据估计全国肺癌发病率和新发患者数。使用统计分析软件SAS 9.0的Genmod模型进行数据分析。

肺癌发病趋势分析采用美国癌症研究所开发的统计软件Joinpoint Regression Program 3.3.1。通过对汇总数据的肺癌发病率对数转换的线性回归分析，计算年度变化百分比（APC）及统计学检验。

## 2 结果

### 2.1 第三次死因回顾抽样调查肺癌的死亡率

第三次死因回顾抽样调查结果显示，样本地区肺癌死亡率为30.83/10万，其中男性41.34/10万，女性19.84/10万；年龄结构调整死亡率分别为20.24/10万、28.60/10万和12.18/10万，均为男性、女性中首位的癌症死亡原因。城市地区肺癌死亡率为40.98/10万，排第一位，明显高于农村地区的26.93/10万。

与前二次死因调查结果比较，死亡率较70年代的5.60/10万和90年代的15.19/10万明显上升。年龄调整死亡率也分别升高了261.43%和33.25%。在癌症死亡中的构成也由7.35%和16.20%提高至22.70%，从第5位和第3位的癌症死因跃居第一位。

肺癌死亡率随年龄增加而增加，城市地区的男性女性在80岁-84岁年龄段死亡率均最高，分别为716.86/10万和318.26/10万。农村地区，男性也是在80岁-84岁年龄段达到最高，为400.06/10万，女性则在85岁以上组最高，为205.92/10万（表 1）。

**1:** 全国第三次死因调查肺癌年龄别死亡率 The age-specific death rate of lung cancer in the national 3rd death survey

Region	Gender	Age-specific death rate (1/100 000)												
		0-29	30-34	35-39	40-44	45-49	50-54	55-59	60-64	65-69	70-74	75-79	80-84	85^+^
Urban areas	Male	0.35	3.64	7.22	18.10	31.12	61.28	97.93	159.12	285.98	452.62	594.60	716.86	618.51
	Female	0.24	1.14	3.90	7.80	15.23	25.96	40.20	66.93	117.72	198.31	261.10	318.26	278.15
Rural areas	Male	0.35	2.62	6.59	13.33	23.80	54.82	93.55	137.53	199.88	300.11	370.37	400.06	340.19
	Female	0.24	1.63	3.99	7.75	10.49	24.18	36.01	55.51	77.84	122.29	150.32	168.38	205.92

### 2.2 中国肺癌发病的估计

全国肿瘤登记中心收集各肿瘤登记处的发病死亡和人口资料。2004年和2005年，共45个肿瘤登记处提供了恶性肿瘤发病和死亡数据，从中选取32个登记处资料较好的进行合并。

利用*Poisson*回归Genmod模型进行拟合，估计地区调整后的肺癌死亡发病比（表 2）。城市农村地区不同年龄死亡发病比均大于0.5，大多在0.7和1之间，可见肺癌的预后较差。各别年龄组死亡发病比大于1，多见老年组，可能跟发病漏报有关系。

**2:** 全国第三次死因调查肺癌年龄别死亡率 The age-specific death rate of lung cancer in the national 3rd death survey

Region	Gender	Age specific mortality incidence ratio												
		0-29	30-34	35-39	40-44	45-49	50-54	55-59	60-64	65-69	70-74	75-79	80-84	85^+^
Urban areas	Male	0.623	0.669 7	0.682 8	0.724	0.772 8	0.714 5	0.808	0.774 3	0.889 6	0.968 6	1.077 7	1.164 5	1.120 2
	Female	0.638 9	0.77	0.752 2	0.625 9	0.654 8	0.661 3	0.745 9	0.788 8	0.915 6	1.059 1	1.175 9	1.333	1.379 1
Rural areas	Male	0.507 8	0.719 2	0.763 3	0.7	0.738 9	0.797 5	0.854 4	0.780 2	0.883 3	0.878 8	1.016 9	0.966 2	0.957 5
	Female	0.999 9	0.592 6	0.732 7	0.642 7	0.770 3	0.705	0.788 8	0.845 9	0.828 5	0.867	1.060 8	1.072 6	1.228 1

根据死亡率和死亡发病比，可计算出全国不同地区不同性别的年龄别发病率（表 3）。然后通过2005年1%人口抽样调查的人口数据估计全国肺癌的新发病例数和死亡数（表 4）。2005年估计全国肺癌新发病例数为536 407人，其中城市地区共264 249（男性182 173人，女性82 076人），农村地区共272 158（男性189 020人，女性83 138人）。全年肺癌死亡病例数为475 768人，其中城市地区共240 420（男性163 526人，女性76 894人），农村地区共235 348（男性162 937人，女性72 411人）。

**3:** 全国估计肺癌年龄别发病率 The estimated incidence rate of lung cancer in China

Region	Gender	Age specific incidence (1/100, 000)												
		0-29	30-34	35-39	40-44	45-49	50-54	55-59	60-64	65-69	70-74	75-79	80-84	85^+^
Urban areas	Male	0.57	5.43	10.58	24.99	40.27	85.77	121.21	205.51	321.47	467.29	551.73	615.60	552.14
	Female	0.37	1.48	5.19	12.46	23.26	39.26	53.90	84.85	128.57	187.24	222.05	238.75	201.69
Rural areas	Male	0.70	3.65	8.63	19.05	32.21	68.74	109.49	176.27	226.29	341.50	364.21	414.05	355.29
	Female	0.24	2.75	5.45	12.06	13.61	34.30	45.65	65.63	93.95	141.04	141.71	156.98	167.67

**4:** 2005年全国估计肺癌新发病例和死亡人数 The total estimated new cases and deaths of lung cancer in China during the year 2005

	Region	Gender	Age specific new cases and deaths													
			0-29	30-34	35-39	40-44	45-49	50-54	55-59	60-64	65-69	70-74	75-79	80-84	85^+^	Total
Deaths	Urban areas	Male	412	1 022	2 170	4 978	6 498	12 547	14 271	16 764	26 100	32 717	26 131	14 817	5 098	1 63 526
		Female	271	328	1 177	2 100	3 126	5 371	5 909	7 143	11 109	14 856	12 500	8 698	4 307	76 894
	Rural areas	Male	552	694	2 133	3 831	5 552	14 984	19 577	21 674	25 669	29 922	22 302	12 042	4 004	162 937
		Female	342	450	1 364	2 334	2 451	6 398	7 010	7 960	9 263	12 519	10 631	7 029	4 661	72 411
New cases	Urban areas	Male	671	1525	3 180	6 873	8 408	17 562	17 664	21 652	29 339	33 777	24 247	12 724	4 551	182 173
		Female	417	426	1 566	3 354	4 774	8 122	7 922	9 055	12 132	14 027	10 631	6 525	3 123	82 076
	Rural areas	Male	1 103	967	2 794	5 475	7 514	18 789	22 913	27 779	29 061	34 048	21 931	12 463	4 182	189 020
		Female	342	760	1 863	3 632	3 180	9 076	8 886	9 411	11 180	14 438	10 022	6 553	3 795	83 138

### 2.3 登记地区肺癌发病率的趋势

1988年-2005年10个肿瘤登记处汇总资料显示男性和女性肺癌粗发病率均有升高，年增长率为1.63%，其中男性为1.30%，女性为2.34%（*P*值均 < 0.05）。而年龄调整发病率略有降低，年变化率为-0.55%，其中男性为-1.05%（*P*值均 < 0.05），女性则无显著改变（图 1）。而发病率变化均在18年中出现转折，对于粗发病率，男女性在2001年以前明显升高，而后趋于稳定；对于年龄调整死亡率，男性在1997年之前变化不明显，而后显著下降；女性则在2001年以前呈上升趋势，此后表现为下降趋势（表 5）。

**1:**
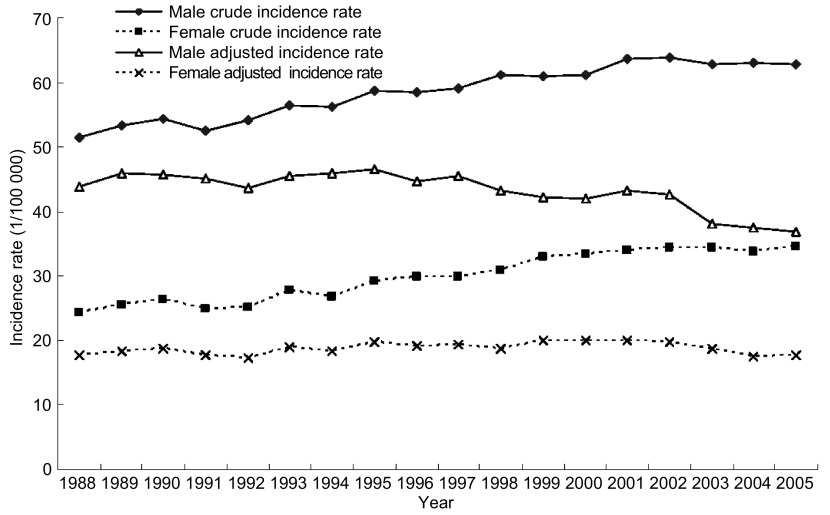
全国10个登记点1988年-2005年肺癌发病率变化趋势 The tendency of the incidence rate for lung cancer in the 10 registries during the year 1988 to 2005

**5:** 全国10个肿瘤登记处肺癌发病率1988-2005年变化百分比分析 Annual percent change (APC) analysis of the incidence rate for lung cancer from 10 cancer registries during the year 1988 to 2005

Incidence rate	Gender	Year	Annual percent change APC (%)	95%confidence interval (%)	*P*
Crude mortalities	Male	1988-2001	1.6	1.3–1.8	< 0.001
		2001-2005	-0.1	-1.6–1.3	0.83
	Female	1988-2001	2.7	2.2–3.2	< 0.001
		2001-2005	0.4	-2.6–3.4	0.80
Age adjusted incidence rate	Male	1988-1997	0.2	-0.7–1.0	0.70
		1997-2005	-2.5	-3.5–-1.5	< 0.001
	Female	1988-2001	1.0	0.5–1.4	< 0.001
		2001-2005	-3.4	-6.2–-0.5	0.03

## 3 讨论

癌症发病的死亡是评价一个国家和地区居民癌症负担，制定防控策略的重要信息。我国自上世纪60年代，在个别地区建立肿瘤登记制度，然而因为种种原因，全国的体系没有很好地建立起来，因为缺乏动态的监测，我国癌症的流行情况不能及时地为卫生行政部门提供可靠信息。因此，我国曾经开展过三次以肿瘤为重点的死因回顾调查，横断面地了解当时我国居民的健康状况和癌症负担情况。同时，全国肿瘤登记中心也在积极加强全人口的肿瘤登记工作。然而，目前我国的肿瘤登记网络尚不完善，登记网络仅覆盖全国5.53%的人口，不能提供具有全国代表性的肿瘤发病数据^[11]^。

2006年，卫生部在全国范围内开展第三次死因回顾抽样调查，目的是了解21世纪初我国城乡、不同类型地区居民以恶性肿瘤为重点的全部死因的死亡率水平、死因构成及其变化趋势，掌握主要恶性肿瘤死亡的地区与人群分布特征，为国家制定疾病预防控制规划、预防保健策略提供依据。调查结果显示，我国肺癌的死亡率增加趋势明显，已经成为第一位的癌症死因，较90年代升高了75.77%，排除年龄结构变化的影响，也增加了33.25%。是我国人群死亡率上升最快的癌症。

吸烟是肺癌病因中最重要的因素。研究^[12]^表明，全球癌症死亡有21%归因于吸烟，其中肺癌影响最大。烟草消费是人类肺癌最主要的可以避免和干预的致癌因素。2002年开展的中国居民营养与健康状况调查^[13]^显示，我国吸烟率为24.0%，其中男性50.2%，女性2.8%，吸烟人数达3.5亿，居世界之首。目前，我国仍没有全国性的控烟运动，肺癌的防治任重道远。此外空气污染与肺癌发病有重要关系。过量摄入油脂、动物脂肪、胆固醇和酒精则增加肺癌的发病风险^[14]^。我国云南个旧锡矿工人中异常高的肺癌发病率与氡子体的电离辐射密切相关^[15]^。职业性接触粉尘的男性吸烟者的全死因、恶性肿瘤（其中50%以上为肺癌）和呼吸系统疾病的协同指数分别为3.16、1.67和2.25，提示职业接触与吸烟间具有协同作用^[16]^。男性肺结核史、呼吸系统疾病史、慢性支气管病史、肺气肿和哮喘史的肺癌相对危险分别是3.1、2.1、1.4和1.3；女性分别是6.1、4.4、1.8和5.3；男性和女性家族肺病史的肺癌发病风险分别为1.9和3.1^[17]^。研究^[14]^表明，在日常饮食中增加新鲜蔬菜和水果的摄入，食用富含胡萝卜素、类胡萝卜素、β-胡萝卜素、α-胡萝卜素、番茄红素、叶黄素、β-玉米黄质、维生素C、维生素E、硒等微量营养素的食物，以及适当体力活动，是降低肺癌发病风险的保护因素。

使用癌症的死亡发病比估计肿瘤负担是常用的统计方法。肿瘤的死亡发病比是评价肿瘤登记数据质量的指标之一。它不仅反映某种癌症的预后，同时反映地区的医疗状况和诊治水平。本研究采用死因调查相同时期的肿瘤登记资料，32个肿瘤登记处的汇总，覆盖人群较多，数据质量经过前过肿瘤登记中心的验证和评估，达到要求，可以较真实反映我国肺癌死亡发病比。研究发现，不同地区不同性别肺癌的年龄别死亡发病比相似，大多在0.7和1之间，说明肺癌的预后比较差，每年的死亡病例接近新发病例。个别年龄组大于1，表明可能存在发病漏报的问题、以及发病延迟、死亡病例在老年组积聚的现象。结合第三次死因回顾抽样调查的肺癌死亡率资料，可以推算出不同地区不同性别的年龄别发病率。结合2005年全国人口抽样调查数据，估计我国2005年肺癌死亡人数475 768例，新发病536 407。男性明显多于女性，城市农村病例数相近。

通过对数据较为齐全的10个肿瘤登记处的汇总资料可以看出，肺癌的发病率自1988年-2005年18年间，以每年1.63%的速度增加，其中男性每年增长1.30%，女性为2.34%。调整年龄结构变化的影响，实际每年以0.55%的速度下降，其中男性每年降低1.05%，女性无明显变化。表明，肺癌发病率的增加主要是由于人口老龄化造成的。

因为我国尚缺乏完善、可靠的全国肿瘤监测数据，部分地区不能代表全国癌症的实际负担和流行趋势，因此建立健全肿瘤登记系统、及时准确反映全国的恶性肿瘤发病、死亡情况是十分必要的。肺癌负担日益加重，大力开展有效的防治措施，尤其是倡导全面戒烟，创建无烟环境的活动，对减轻我国居民的肺癌危害势在必行。
